# A Training School for Nurses

**Published:** 1881-10

**Authors:** 


A Training School for Nurses has been established in connection with the Cincinnati Hospital. The candidates are to be educated in all the branches of medicine including practice, surgery and obstetrics; are to attend all the lectures at the hospital, and are to receive certificates of competency or diplomas as testimonials of their efficiency and capacity.

